# Quantum sensing of weak radio-frequency signals by pulsed Mollow absorption spectroscopy

**DOI:** 10.1038/s41467-017-01158-3

**Published:** 2017-10-17

**Authors:** T. Joas, A. M. Waeber, G. Braunbeck, F. Reinhard

**Affiliations:** Walter Schottky Institut and Physik-Department, Technische Universität München, Am Coulombwall 4, 85748 Garching, Germany

## Abstract

Quantum sensors—qubits sensitive to external fields—have become powerful detectors for various small acoustic and electromagnetic fields. A major key to their success have been dynamical decoupling protocols which enhance sensitivity to weak oscillating (AC) signals. Currently, those methods are limited to signal frequencies below a few MHz. Here we harness a quantum-optical effect, the Mollow triplet splitting of a strongly driven two-level system, to overcome this limitation. We microscopically understand this effect as a pulsed dynamical decoupling protocol and find that it enables sensitive detection of fields close to the driven transition. Employing a nitrogen-vacancy center, we detect GHz microwave fields with a signal strength (Rabi frequency) below the current detection limit, which is set by the center’s spectral linewidth $1{\rm{/}}T_2^*$. Pushing detection sensitivity to the much lower 1/*T*_2_ limit, this scheme could enable various applications, most prominently coherent coupling to single phonons and microwave photons.

## Abstract (web summary)

Dynamical decoupling protocols can enhance the sensitivity of quantum sensors but this is limited to signal frequencies below a few MHz. Here, Joas et al. use the Mollow triplet splitting in a nitrogen-vacancy centre to overcome this limitation, enabling sensitive detection of signals in the GHz range.

## Introduction

Sensitive detectors for weak radio-frequency (>100 MHz) signals of electric, magnetic, or pressure fields would shift several frontiers of physics. They could advance the exploration of phonons on the single-particle level and reveal weak microwave signals encountered in quantum information processing, biomedical imaging, or more exotically, the search for extraterrestrial intelligence^1^.

Driven by this perspective, the past decade has seen the rise of detectors based on Rydberg atoms^2, 3^, superconducting quantum circuits^4–8^, or optomechanical sensors^9–11^. All approaches have achieved noise levels below 10 photons (noise temperatures below 100 mK), an order of magnitude better than state-of-the-art semiconductor detectors^12^ and maser amplifiers^13–16^. However, this performance is only reached in sophisticated setups (Rydberg atoms) or at sub-Kelvin temperatures (optomechanics, superconductors).

**Fig. 1:**
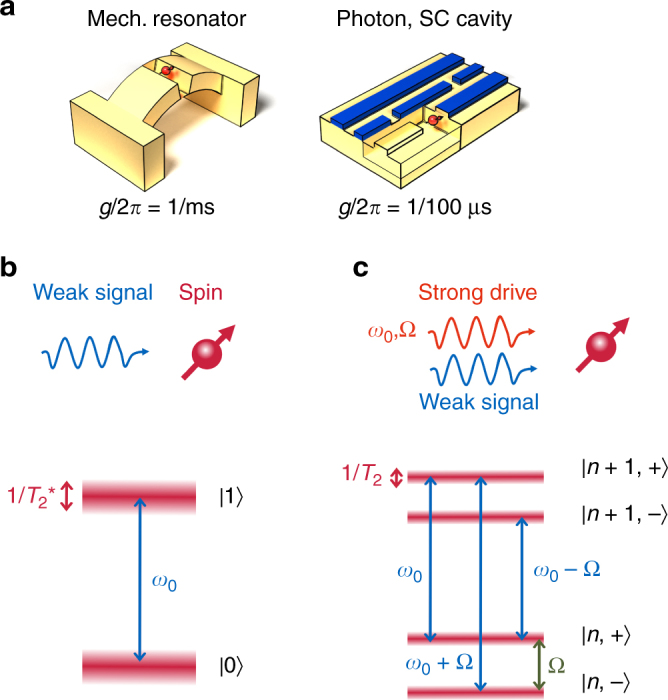
High-frequency sensing by Mollow resonance spectroscopy. **a** Examples of signals which could be coupled to spins within *T*_2_, but which are out of reach of current $T_2^*$-limited protocols^17, 18^ (*g* coupling strength, SC superconductor). **b** High-frequency signals driving the bare spin transition have to be stronger than the inhomogeneous linewidth $1{\rm{/}}T_2^*$. **c** Dressing of the spin by a strong drive creates a set of narrow transitions between new eigenstates described by the photon occupation *n* and the qubit state $\left| \pm \right\rangle = \left( {\left| 0 \right\rangle \pm \left| 1 \right\rangle } \right){\rm{/}}\sqrt 2$ with transition frequencies *ω*_0_, *ω*_0_ ± Ω, which enable *T*_2_-limited sensing

Detectors based on solid state spin qubits could potentially overcome these limitations. Optically active spin qubits such as nitrogen-vacancy (NV) centers can be optically polarized, that is effectively laser cooled to a temperature of a few 10 mK, even in a substrate at higher temperature. Magnetic tuning of their spin transition enables resonant coupling to external fields at any frequency up to 100 GHz. Theory proposals (Fig. 1a) suggest that both single microwave phonons^17^ and photons^18^ can be coupled sufficiently strong to drive a full spin-flip within the spin coherence time *T*_2_ (ms^19^ to s^20^, depending on species and temperature).

However, radio-frequency sensing by spin qubits is currently precluded by a major roadblock. It is illustrated in the detection protocol of Fig. 1b, where an incoming signal drives the qubit transition, inducing a spin flip which is subsequently detected by readout of the spin. To drive a full spin-flip, an incoming signal has to saturate the spin transition. Therefore, the signal strength (Rabi frequency) has to exceed the inhomogeneous transition linewidth ${\rm{\Delta }}\omega \sim 1{\rm{/}}T_2^*$. Since $1{\rm{/}}T_2^*$ is much broader than 1/*T*_2_ (MHz vs kHz for an NV center in a natural abundance crystal at room temperature), coupling of spins to high-frequency signals remains inefficient. As a specific example, interfacing spins to single phonons or photons (Fig. 1a) is currently precluded, since coupling would be possible within *T*_2_ but remains out of reach of $T_2^*$.

For signal frequencies below a few MHz, dynamical decoupling protocols can break this limit^21^ (Fig. 1c). Here, the transition is driven by a strong continuous or pulsed control field (frequency *ω*_0_) to create a pair of photon-dressed qubit states, split by the driving field Rabi frequency Ω^22, 23^. This new transition has a far narrower linewidth 1/*T*_2_, and can hence absorb much weaker signals. However, practical limitations on drive power limit the frequency range which can be probed in this way.

As the key idea of this work, we note that the fully hybridized spin-photon states (the ‘Jaynes-Cummings ladder’) support another set of transitions at frequencies (*ω*_0_ − Ω, *ω*_0_, *ω*_0_ + Ω), the Mollow triplet^24^, which has been extensively studied in quantum optics^25–29^ and has been proposed as a narrowband tunable photon source^30^. Since these transitions equally link pairs of dressed states, we posit that they should allow for *T*_2_-limited sensing of signals with a frequency much higher than the available Rabi frequency Ω. We will show in the following that these transitions can indeed be harnessed to detect high-frequency signals. Moreover, we will demonstrate that Mollow sidebands can be created by robust dynamical decoupling protocols acting as a strong drive. In contrast to a continuous drive, these protocols set the effective Rabi frequency by timing rather than power, an experimental advantage that has made low-frequency sensing by decoupling a widely adopted technique. We will analyze the sensitivity of the resulting schemes, concluding that they could enable coherent coupling of solid state spins to single phonons and photons.

## Results

### Continuous wave Mollow absorption

**Fig. 2:**
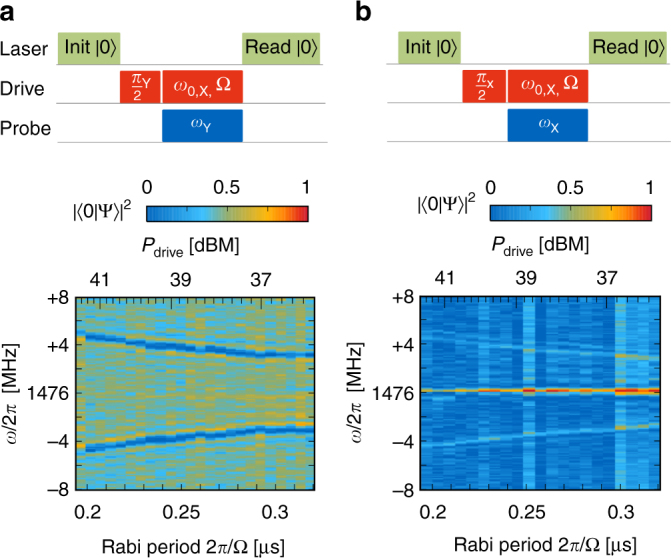
Mollow absorption. **a** A weak probe with phase orthogonal to a strong drive field drives the sideband transition at *ω* = *ω*_0_ ± Ω. Measurements were performed on an NV center with optical initialization and readout (green laser pulses). The duration of the drive (~1.3 μs) was adjusted for every drive field power Ω (set by the microwave power *P*_drive_) to correspond to an integer number of *π* rotations. **b** A weak probe in phase with the strong drive field drives the central transition *ω*_0_

We demonstrate the creation of dressed states by the scheme of Fig. 2a. Here, the spin is initialized into the dressed state $\left| + \right\rangle = \left( {\left| 0 \right\rangle + \left| 1 \right\rangle } \right){\rm{/}}\sqrt 2$ by a (*π*/2)_Y_ pulse (Y labeling the carrier phase *ϕ*_Y_ = *π*/2). This state is locked as an eigenstate of a strong dressing field with orthogonal carrier phase *ϕ*_*X*_ = 0. We find that a weak probe field at the detuned frequency *ω*_0_ ± Ω indeed induces rotation at its native Rabi frequency, as evidenced by measurements on an NV center. The central Mollow resonance is absent in this measurement, since it couples dressed states with identical spin projection, as has been previously observed in superconducting qubits^31^. It can be recovered by preparing into an orthogonal state and changing the phase of the signal (Fig. 2b) to account for the different quadratures of the Mollow sidebands^25^.

### Pulsed Mollow absorption as a sensing protocol

**Fig. 3:**
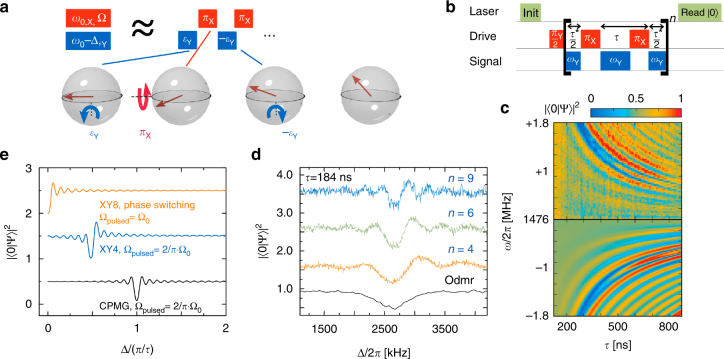
Pulsed Mollow absorption spectroscopy (subfigures ordered clockwise). **a** The sideband transitions of the CW protocol in Fig. 2a can be understood as a dynamical decoupling protocol by dissecting the strong drive into a train of *π* pulses and the probe into a train of weak pulses ($\epsilon$). The detuning Δ of the probe translates into periodic inversions of its axis, which are resonantly rectified by the strong drive. **b** Pulse sequence for high-frequency sensing, a direct implementation of the interpretation given in **a**. *π* pulses at frequency *ω*_0_ emulate a strong drive to resonantly enhance a weak signal at frequency *ω* = *ω*_0_ + Δ. The pulse spacing *τ* incorporates the duration of a *π* pulse. *τ* ^*^ is obtained from *τ* by subtracting the *π* pulse duration. **c** Pulsed Mollow resonance, as measured on a NV center (upper plot) and simulated (lower plot). A resonance at Δ = *π*/*τ* is framed by sidebands with nodes at Δ = ± *π*/*τ* ± *kπ*/*T* (with $k \in {\Bbb N}$, sequence duration *T*=*2nτ*, cycle number *n* = 4). **d** Linewidth of the resonance. The line narrows below the natural linewidth ${\rm{1/}}T_2^*$ (as observed in an optically detected magnetic resonance (odmr) experiment, see also Supplementary Note 1) for sequences longer than the dephasing $\left( {T  >T_2^*} \right)$. **e** Simulated spectral response to sensing sequences with different decoupling protocols. The pulse spacing between 24 *π* pulses was kept constant at *τ* = 127.6 ns. The stated effective Rabi frequencies are for Rabi oscillations driven on the Mollow resonance. ‘XY8, phase switching’ refers to the protocol of Fig. 4. More detailed discussion in Supplementary Note 4. Data traces in **d**, **e** have been shifted vertically for better comparison by an offset of 1.0. The ODMR trace in **d** is shifted horizontally by 2623 kHz

We now convert Mollow absorption spectroscopy into a pulsed sensing protocol (Fig. 3a) to mitigate an important problem: the continuous wave (CW) protocol is prone to decoherence since fluctuations of the drive field power (Ω) directly translate into frequency noise of the dressed state transition *ω*_0_ ± Ω. We will see that pulsed protocols shift the frequency of the Mollow sideband absorption from *ω*_0_ ± Ω to *ω*_0_ ± *π*/*τ*, *τ* denoting the pulse spacing as shown in Fig. 3b. Since timing (*τ*) is controlled better than power (Ω), decoherence is reduced to the intrinsic limit set by the spin qubit. Importantly, absorption on these transitions will induce a spin trajectory similar to standard Rabi oscillations. This enables sensing of the absorbed field’s amplitude, effectively turning the probe into a signal field.

Conversion into a pulsed protocol is best understood from tracking the spin evolution across the sideband absorption sequence (Fig. 3a). We decompose the strong drive into a series of *π* pulses, spaced by a time *τ* = *π*/Ω, and split the weak signal into a commensurate series of weak pulses with pulse area ${\it{\epsilon }} \ll \pi$. We equally discretize its detuning of Δ = Ω − a continuous decrease in carrier phase-into periodic inversions of its axis, that is a discrete decrease of the phase by *π* occurring with period *π*/Δ. At the resonance condition Δ = Ω, this period matches the spacing *τ* = *π*/Ω of the strong drive. In this case, the weak signal is resonantly rectified in the toggling frame of the spin (Fig. 3a), analogous to the situation in low-frequency sensing. We note that discretization preserves the axes of all fields involved up to a sign so that the resulting absorption resonance remains phase-sensitive. While it picks up signals with a carrier phase along Y, it is blind to signals along X.

Our pulsed scheme (Fig. 3b) is an explicit implementation of this discretized sequence. We emulate the strong drive with Rabi frequency Ω by short *π* pulses with a spacing *τ* = *π*/Ω. We equally discretize the amplitude of the signal into a sequence of pulses, applied between the *π* pulses of the strong drive. We do not discretize its phase, allowing instead for a continuous detuning Δ, since this property should be sufficient to induce the advance in carrier phase discussed above. We find that the weak signal is most strongly absorbed at a detuning Δ/2π = ±(2*τ*)^−1^. The absorption resonance remains locked to this frequency as we scan *τ* while keeping all other parameters constant (Fig. 3c). All our observations match well with an explicit time domain simulation of the spin evolution (bottom half of Fig. 3c, Supplementary Note 3). We note that discretization (switching) of the signal is not strictly required. All of the above analysis remains valid for a continuous signal, despite the fact that it overlaps with the control pulses during part of the time. We have confirmed this prediction experimentally (Supplementary Note 5), but stick with the switched implementation in the following, since it allows for easier implementation of more robust decoupling sequences (see below).

The bandwidth *Δω* = *π*/*T* of this pulsed Mollow resonance is limited by the finite duration *T* = 2*nτ* of the sequence containing 2*n π*-pulses. Crucially, this bandwidth drops below the inhomogeneous linewidth $1{\rm{/}}T_2^*$ if we choose a sequence longer than $T_2^*$ (Fig. 3d). The Mollow resonance is framed by sidebands with nodes at frequencies *ω*_0_ ± *π*/*τ* ± *kπ*/*T* with $k \in {\Bbb N}$. These are another consequence of the finite sequence length: since sensitivity is nonzero only in a rectangular window in the time domain, the sequence has a sinc response in the frequency domain. Tracing a Rabi oscillation of the weak signal along the resonance hyperbola Δ = ± *π*/*τ* we find its native Rabi frequency Ω_rf_ to be reduced to a value Ω_pulsed_ = 2/*π* · Ω_rf_. We attribute this reduction to the fact that the detuned signal, rotating at an angular frequency of Δ on the Bloch sphere, has a phase orthogonal to the strong drive phase only during a fraction of the free evolution time *τ*. It therefore has to be scaled by a factor $\left( {{\int}_0^\tau {{\rm{sin}}\left( {\phi (t)} \right){\rm d}t} } \right){\rm{/}}\tau$, with *ϕ*(*t*) denoting the phase of the signal. All of these properties are analogous to similar features in low-frequency decoupling sequences^32^.

Using the time domain simulation and an analytical model (Supplementary Notes 2 and 4), we find Mollow resonances in many decoupling sequences, including the robust sequences CPMG, XY4, and XY8 (Fig. 3e). A detailed discussion of the effect of decoupling sequence structure on position and shape of the resonances is given in Supplementary Note 4.

### Small-signal limit and impact of decoherence

**Fig. 4:**
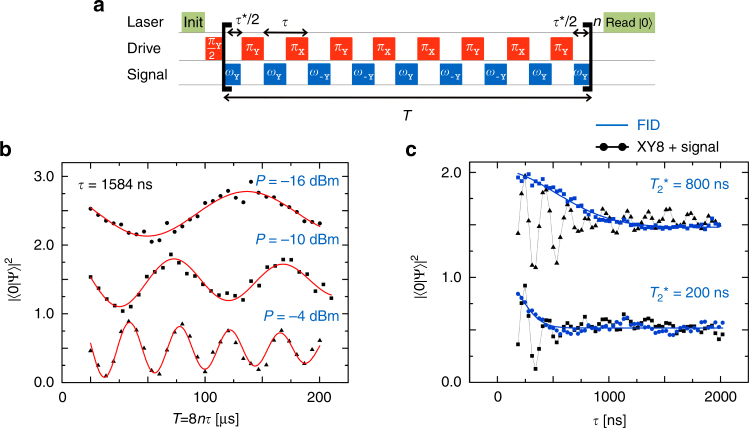
Performance and limits of the method. **a** XY8 sequence employed to record the data of **b**, **c**. The signal was phase-cycled in order to maximize sensitivity. **b** Rabi oscillations much slower than $T_2^{\boldsymbol{*}}$ = 1.8 μs, where the number of *π* pulses was increased up to 120 while keeping the pulse spacing *τ* and the signal power P constant. **c** Signal decay for increasing pulse spacing. For a spacing $\tau  >T_2^{\boldsymbol{*}}$, the pulsed Mollow protocol loses sensitivity. Data traces in **b**, **c** have been shifted vertically by an offset of 1.0 for better comparison

We finally demonstrate the performance and an important limit of our method by the protocol of Fig. 4a. Here, we adopt the XY8 sequence for the strong drive, in order to be maximally robust against experimental fluctuations. We phase-modulate the signal to gain a constructive contribution to the Rabi rotation during every evolution period *τ* ^∗^. In this setting we have been able to drive slow Rabi oscillations with a period as long as 100 µs (Fig. 4b). While this clearly breaks the $T_2^*$ limit in terms of signal strength, the limit reappears as a constraint on the pulse spacing *τ*, which has to be short against $T_2^*$. For longer spacings—corresponding to slower Rabi frequencies in the CW sequence—the Mollow resonance merges with the inhomogeneously broadened transition. To verify this limit explicitly, we artificially shorten $T_2^*$ of the NV center by averaging multiple measurements taken at different, Gaussian-distributed, frequencies of the microwave drive. Tuning decoherence by this technique, we find that sensitivity breaks down if pulses are spaced by more than $T_2^*$ (Fig. 4c).

## Discussion

With these insights, we are finally in a position to evaluate the sensitivity that could be reached by a microwave spin sensor. Table 1 presents a series of such estimates for three typical experimental scenarios, a single NV center at ambient temperature, a NV center at cryogenic temperature with single shot readout, and an ensemble of NV centers in a densely doped diamond. Our estimates derive from two assumptions:

1. We assume high-frequency sensing to be as robust against experimental fluctuations as low-frequency sensing, since it is based on the very same decoupling protocols. In particular, we assume that the same *T*_2_ time can be reached and the same number of control pulses can be applied. This assumption is justified since sensitivity characterizes the response to an infinitesimally weak signal where the spin follows a nearly identical trajectory as in the bare decoupling sequence.
2. In contrast to low-frequency sensing, *T*_2_ is bounded by an upper limit of ${N_{{\rm{max}}}} \cdot T_2^*$, where *N*_max_ ≈ 1000 denotes the maximum number of control pulses that can be applied before pulse errors deteriorate coherence^19^. This condition arises from the additional constraint that pulses have to be spaced by less than $T_2^*$, as discussed in the context of Fig. 4. While this condition does not set the limit for experiments on single NV centers, where isotopic purification can push $T_2^*$ times into the range of 100 µs, it is the limiting factor for ensemble sensing where inhomogeneous broadening shortens $T_2^*$ times down to the sub-microsecond timescale.

**Table 1:** Sensitivity of a NV-based sensor for high-frequency signals

	Single NV, 300 K	Single NV, < 77 K, ^12^C, SSR	NV Ensemble, 300 K
*T*_2_	1 ms^21^	10 ms^19^	100 µs
$T_2^*$	2 µs	100 µs^36^	100 ns^37^
#Pulses	> 500^19^	> 100^19^	1000^19^
#NV	1	1	10^11^ ^38^
Spin detection efficiency *σ*	0.1%^21^	100%^39^	0.1%^21^
Sensitivity *η*	5 nT/$\sqrt {{\rm{Hz}}}$ ^40^	50 pT/$\sqrt {{\rm{Hz}}}$	1 pT/$\sqrt {{\rm{Hz}}}$ ^38^

< 77 K refers to cryogenic conditions, ^12^C to isotopically pure diamond, and SSR to single shot readout. Sensitivities have been estimated as $\eta = 1{\rm{/}}\left[ {2\pi \gamma } \right.\sqrt {\left. {\sigma {T_2}{N_{{\rm{NV}}}}} \right]}$ where *γ* denotes the gyromagnetic ratio, *σ* the spin detection efficiency and *N*_NV_ the number of NV centers. References refer to published values for the respective parameter. Parameters without reference are computed from the others or have been measured in this work.

More importantly, these estimates suggest that NV centers should be able to couple coherently to photons and phonons in the scenarios of Fig. 1 within their coherence time *T*_2_ (assuming the values of Table 1). This would enable detection of both particles by coherent absorption and subsequent detection of the spin state, a more powerful measurement than time-averaged detection of a signal with a mean strength on the single-particle level. It could pave the way to a quantum bus based on these signals, mediating coupling between distant spins or to other qubits. The narrow transition provided by our scheme could aid the development of room-temperature MASERs based on optically initialized spins^14^. Their use as amplifiers could provide another approach to sensing of weak signals, complementary to optical detection.

In summary, we have pushed spin-based quantum sensing to frequencies much higher than the available Rabi frequency Ω. In the language of superconducting amplifiers, this promotes spins to phase-sensitive microwave detectors that might provide sufficient sensitivity to detect single phonons and photons. Compared to competing approaches such as Josephson parametric amplifiers, our scheme has a very narrow bandwidth. It absorbs signals only within a narrow window of width 1/*T*_2_, (≈100 Hz–10 kHz for NV centers) and, operated as a detector, would be limited to a maximum count rate of the same order of magnitude. It seems plausible, however, that a future extension of our experiment could continuously shift this window across frequencies up to several 100 GHz, tuning the spin transition e.g. by a magnetic field^33^. Crucially, the absorption frequency *ω*_0_ ± *π*/*τ* is set only by timing and frequency of the external drive, which can be controlled well. It is independent of the native spin transition and hence resilient to drifts in surrounding fields.

From a fundamental perspective, we have provided an intuitive microscopic understanding of the Mollow triplet as a pulsed quantum protocol. It appears most intriguing to extend this novel perspective to other effects of quantum interference, such as electromagnetically induced transparency.

## Methods

### NV center preparation

All experiments have been performed on single NV centers spontaneously created inside a polycrystalline electronic grade IIa diamond during chemical vapor deposition (Element Six, part N° 145-500-0356).

### Quantum control

Both the strong drive and the weak signal were generated by an Arbitrary Waveform Generator (Rigol DG5352), which was mixed onto a GHz frequency carrier, amplified (amplifier MiniCircuits ZHL16W-43-S+), and applied to the NV center by a coplanar waveguide. All given microwave excitation powers refer to the input of the coplanar waveguide. They have been calculated from the output power of the Arbitrary Waveform Generator by adding a constant offset of +56 dBm to account for all gains and losses along the excitation path.

### Spin readout

The spin state was measured by fluorescence readout in a high-NA confocal microscope (excitation 532 nm, ~1 mW power, detection in the > 650 nm band by an objective lens Olympus UPLSAPO 60 × 1.35O). In total, 4–8×10^5^ readout repetitions per trace were made, corresponding to a measurement time of 15–30 min for each trace. All sequences were recorded twice, with and without an additional *π* pulse before readout. The difference of both datasets was normalized to the signal contrast of a Rabi oscillation to yield a quantitative estimate of ${\left| {\left\langle {0\left| \psi \right.} \right\rangle } \right|^2}$.

### Data availability

All relevant data is available from the authors upon request.

## Electronic supplementary material

Supplementary Information
