# Supplementary material for: Quantum sensing of weak radio-frequency signals by pulsed Mollow absorption spectroscopy
Source: Nat Commun. 2017 Oct 17;8:964. doi: 10.1038/s41467-017-01158-3 (PMC5645369; doi:10.1038/s41467-017-01158-3)
Supplement: Supplementary file 1 — Supplementary Information [file 41467_2017_1158_MOESM1_ESM.pdf]

Supplementary Note 1: Natural qubit transition linewidth

The natural linewidth of a qubit transition is determined by the qubit dephasing time as $\Delta f_{\mathrm{FWHM}} = 1/T_2^*$. However, the measured linewidth in an optically detected magnetic resonance (ODMR) experiment does not necessarily reflect the natural limit as several mechanisms, predominantly power broadening owing to the finite pulse bandwidth, can significantly broaden the resonance. In order to verify that the ODMR spectrum shown in Fig. 3d of the main text is not subject to parasitic broadening we test the consistency of the measured linewidth with a free induction decay (FID) experiment. As the FID is inherently unaffected by power broadening it provides a more reliable measure of the dephasing time.

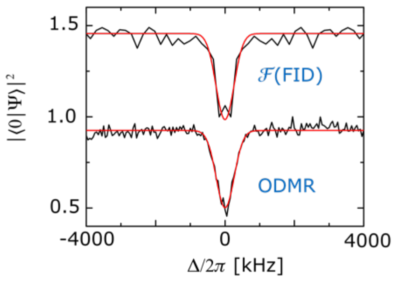

Supplementary Figure 1 - Linewidth of the ODMR resonance. The black bottom trace is a reproduction of the ODMR curve shown in Fig. 3d of the main text. The signal amplitude was normalized to the maximum Rabi signal contrast. A Fourier transform of a time-domain FID measurement is shown in the upper trace (amplitude multiplied by 0.5 and shifted vertically by 1.0 for clarity). Red curves show fits with a Gaussian peak profile.

Supplementary Figure 1 shows the direct comparison of the Fourier transformed FID data with the measured ODMR curve. Gaussian fits to the resonance dips (red lines) yield

$$\Delta f_{\mathrm{FWHM,FID}} = (790 \pm 50)\ \mathrm{kHz} \quad (1)$$

$$\Delta f_{\mathrm{FWHM,ODMR}} = (610 \pm 14)\ \mathrm{kHz} \quad (2)$$

We conclude that the ODMR linewidth is in qualitative agreement with the FID curve. The linewidth of the Mollow sideband resonances shown in Fig. 3d is therefore below the natural linewidth, confirming the expectation that our sensing protocol is not limited by T_2^*.

Supplementary Note 2: Description of Mollow spectra by a sensitivity function

We now derive an analytical model to predict the sensitivity of a dynamical decoupling sequence to small high-frequency signals $\Omega_{\mathrm{x}}(t)\boldsymbol{\sigma}_{\mathbf{x}} + \Omega_{\mathrm{y}}(t)\boldsymbol{\sigma}_{\mathbf{y}}$ of arbitrary axis (carrier phase) X or Y (Supplementary Figure 2a-b). We do this by transferring the sensitivity function formalism established for low-frequency sensing. It states that a weak signal field $\Omega_{\mathrm{z}}(t)$ applied along a dynamical decoupling sequence induces a spin signal $\langle\psi|\boldsymbol{\sigma}_{\mathbf{z}}|\psi\rangle = \int g(t)\Omega_{\mathrm{z}}(t)dt$. Here, $g(t)$ is the **sensitivity function,** a function $g(t)\colon \mathbb{R} \to \{1,-1\}$ quantifying the impact of an infinitesimal signal Ω_{z} applied at time t on the final measurement outcome. This formalism can be extended to high-frequency sensing, with three main results

- High-frequency sensing is insensitive to signals with a carrier phase along axis X.
- The impact of signals along carrier phase Y can be quantified by projection onto a sensitivity function $g_{\mathrm{Mollow}}(t)\colon \mathbb{R} \to \{1,-1\}$ according to the relation $\langle\psi|\boldsymbol{\sigma}_{\mathbf{z}}|\psi\rangle = \int g_{\mathrm{Mollow}}(t)\Omega_{\mathrm{y}}(t)dt$
- $g_{\mathrm{Mollow}}(t)\colon \mathbb{R} \to \{1,-1\}$ is a piecewise constant function. It changes sign at every π pulse applied along X. It is left invariant by a π pulse along Y, which is in contrast to low-frequency sensing.

Without loss of generality, all these statements refer to a sequence where the initial $\pi/2$ pulse is applied along axis Y.

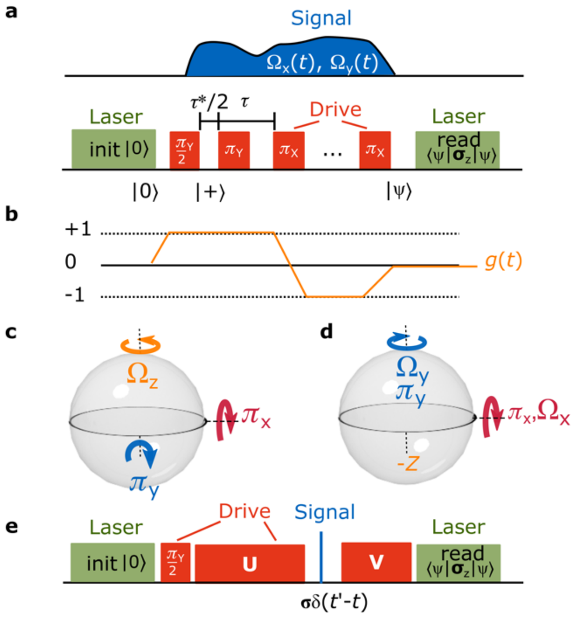

Supplementary Figure 2 – Sensitivity function formalism. (a) We consider high-frequency sensing of a weak signal field $\Omega_{\mathrm{x}}(t), \Omega_{\mathrm{y}}(t)$ by a dynamical decoupling protocol involving π pulses along axes X and Y, corresponding to orthogonal carrier phases. As in the main text, τ^*refers to the free evolution time, defined as pulse spacing τ minus the duration of a π pulse. (b) The measurement outcome $\langle\psi|\sigma_{\mathrm{z}}|\psi\rangle$ can be computed as $\langle\psi|\sigma_{\mathrm{z}}|\psi\rangle = \int g_{\mathrm{Mollow}}(t)\Omega_{\mathrm{y}}(t)dt$. Here g(t) is a sensitivity function modeling the change of measurement outcome induced by an infinitesimal signal applied at time t. It changes sign with every π pulse along axis X and maintains sign across π pulses along Y. (c,d) Low-frequency sensing (c) is equivalent to high-frequency sensing (d) by the identification $Z' \to Y, Y' \to -Z$. (e) Model used in the derivation. π pulses before and after an infinitesimal signal at time t are grouped to operators U and V.

To provide a physical picture, we first review the situation of low-frequency dynamical decoupling (Supplementary Figure 2c). Here, the spin is prepared in a coherent superposition and repeatedly flipped by π pulses along axes with orthogonal carrier phases X and Y. The phase imprinted onto a superposition state $(|0\rangle + e^{i\phi}\,|1\rangle)/\sqrt{2}$ by a time-varying drive Ω_z is computed as $\phi = \int g(t)\Omega_z(t)dt$. Here, the sensitivity function *g(t)* changes sign with every π pulse, regardless of the

pulse axis. It models the fact that Ω_z changes sign with every π pulse in the toggling frame of the spin. The measurement outcome $\langle\psi|\boldsymbol{\sigma_z}|\psi\rangle$ is roughly identical to ϕ for small signals. $\langle\psi|\boldsymbol{\sigma_z}|\psi\rangle = \sin(\phi) \approx \phi$.

High-frequency sensing (Supplementary Figure 2d) can be mapped to this situation by rotating the Bloch sphere by $\pi/2$ around axis X, identifying axes Z' with Y and Y' with –Z, respectively. Furthermore, we note that the component Ω_x of the signal induces no rotation in the limit of a very weak signal. Here, the spin never evolves far away from the axis X, remaining approximately in the eigenstates $|+\rangle, |-\rangle$ of the spin operator $\boldsymbol{\sigma_\mathrm{x}}$. In the toggling frame, the only relevant component, Ω_Y, is left invariant by π pulses along Y, but changes sign with every π pulse along X. This can be modeled by a sensitivity function $g_\mathrm{Mollow}(t)$ with the same properties.

The relation $\langle\psi|\boldsymbol{\sigma_z}|\psi\rangle = \int g_\mathrm{Mollow}(t)\Omega_\mathrm{y}(t)dt$ can equally be derived analytically. The sensitivity of the measurement outcome $\langle\psi|\sigma_\mathrm{z}|\psi\rangle$ to an infinitesimal signal applied at time t along an axis $\boldsymbol{\sigma}$ can be formally computed as the functional derivative

$$g_\mathrm{Mollow}(t) = \frac{d\langle\psi|\sigma_\mathrm{z}|\psi\rangle}{d\,\boldsymbol{\sigma}\delta(t'-t)} = \langle\psi|\sigma_\mathrm{z}\cdot\frac{d|\psi\rangle}{d\,\boldsymbol{\sigma}\delta(t'-t)}\cdot + H.C. \qquad (3)$$

Grouping the control pulses before and after the infinitesimal signal to operators $\boldsymbol{U}, \boldsymbol{V}$ as illustrated in Supplementary Figure 2e, we can rewrite the derivative of the final state as

$$\frac{d|\psi\rangle}{d\,\boldsymbol{\sigma}\delta(t'-t)} = -i\boldsymbol{V}\boldsymbol{\sigma}\boldsymbol{U}|\psi\rangle \qquad (4)$$

We analyze this term separately for signals along $\boldsymbol{\sigma} = \boldsymbol{\sigma_\mathrm{x}}$ and $\boldsymbol{\sigma} = \boldsymbol{\sigma_\mathrm{y}}$.

The sequence is insensitive to signals along $\boldsymbol{\sigma_\mathrm{x}}$. To see this, we note that signals are small. Therefore, the spin state $|\psi\rangle$ always remains approximately in an eigenstate of $\boldsymbol{\sigma_\mathrm{x}}$. $|\psi\rangle = |+\rangle = (|0\rangle + |1\rangle)/\sqrt{2}$ or $|\psi\rangle = |-\rangle = (|0\rangle - |1\rangle)/\sqrt{2}$, regardless of the π pulses applied. The action of $\boldsymbol{\sigma}$ in Eq. (4) reduces to a multiplication with ± 1, which does not alter the spin state. Consequently, $\langle\psi|\boldsymbol{\sigma}_\mathrm{z}\cdot\frac{d|\psi\rangle}{d\,\boldsymbol{\sigma_\mathrm{x}}\delta(t'-t)} = 0$, since all matrix elements of $\boldsymbol{\sigma_\mathrm{z}}$ involving equal spin states vanish: $\langle-|\boldsymbol{\sigma_\mathrm{z}}|-\rangle = 0, \langle+|\sigma_\mathrm{z}|+\rangle=0$.

In contrast, signals along $\boldsymbol{\sigma_\mathrm{y}}$ do alter the spin state. Their influence on the measurement outcome $\langle\psi|\boldsymbol{\sigma_z}|\psi\rangle$ depends on their position in the sequence and changes sign with every π pulse along X. To see this, we consider the cases that the infinitesimal signal is followed by a π pulse along Y or X, respectively. Due to the anticommutation relation $\{\boldsymbol{\sigma_\mathrm{y}}, \boldsymbol{\sigma_i}\} = \delta_{\mathrm{y}i}$, a signal along axis Y commutes with a π pulse along Y. It anticommutes with a π pulse along axis X so that it has opposite effects before and after a π_x pulse. Accordingly, the sensitivity function $g_\mathrm{Mollow}(t)$ has to maintain its value across a π pulse along Y, and has to change sign with every π pulse along X.

Supplementary Note 3: Time domain simulations

All simulations are based on the semi-classical Rabi Hamiltonian

$$\hat{H} = \frac{\hbar}{2}(\mathit{\Delta}\boldsymbol{\sigma}_\mathbf{z} + \mathit{\Omega}\left(\cos(\phi)\,\boldsymbol{\sigma}_\mathbf{x} + \sin(\phi)\,\boldsymbol{\sigma}_\mathbf{y}\right)) \tag{5}$$

where $\mathit{\Delta} = \omega_0 - \omega$ is the detuning of the resonant qubit two-level transition ω_0 from the coupled electromagnetic field with frequency ω, phase ϕ and resonant Rabi frequency $\mathit{\Omega}$.

For every pulse in the sequence we calculate the time evolution operator, which is determined by the unified Rabi frequency $\hat{\mathit{\Omega}}_\mathrm{u}$.

$$\hat{U} = \exp\left(-\frac{i}{2}\hat{\mathit{\Omega}}_\mathrm{u}\,t\right) \tag{6}$$

If only one electromagnetic field couples to the two-level system, the well-known expression for the unified Rabi frequency in rotating wave approximation is

$$\hat{\mathit{\Omega}}_\mathrm{u}^* = \mathit{\Omega}\cos(\phi)\,\boldsymbol{\sigma}_\mathbf{x} + \mathit{\Omega}\sin(\phi)\,\boldsymbol{\sigma}_\mathbf{y} + \mathit{\Delta}\boldsymbol{\sigma}_\mathbf{z} \tag{7}$$

However, in our setting two fields must be considered. The weak signal field ω_rf is detuned from the spin transition by $\mathit{\Delta}_\mathrm{rf} = \omega_0 - \omega_\mathrm{rf}$. In addition, we have a resonant dressing field which correspondingly is detuned from the weak field by $\mathit{\Delta}_\mathrm{rf,dd} = \omega_\mathrm{rf} - \omega_\mathrm{dd}$ (where generally $\omega_\mathrm{dd} \approx \omega_0$ and hence $\mathit{\Delta}_\mathrm{rf,dd} \approx -\mathit{\Delta}_\mathrm{rf}$).

For practical purposes, we now switch to the rotating frame of the weak signal field where the respective expressions are

$$\hat{\mathit{\Omega}}_\mathrm{rf} = \mathit{\Omega}_\mathrm{rf}\cos(\phi_\mathrm{rf})\,\boldsymbol{\sigma}_\mathbf{x} + \mathit{\Omega}_\mathrm{rf}\sin(\phi_\mathrm{rf})\,\boldsymbol{\sigma}_\mathbf{y} + \mathit{\Delta}_\mathrm{rf}\boldsymbol{\sigma}_\mathbf{z} \tag{8}$$

$$\hat{\mathit{\Omega}}_\mathrm{dd} = \mathit{\Omega}_\mathrm{dd}\cos\left(\phi_\mathrm{dd} + \mathit{\Delta}_\mathrm{rf,dd}t\right)\boldsymbol{\sigma}_\mathbf{x} + \mathit{\Omega}_\mathrm{dd}\sin\left(\phi_\mathrm{rf} + \mathit{\Delta}_\mathrm{rf,dd}t\right)\boldsymbol{\sigma}_\mathbf{y} + \mathit{\Delta}_\mathrm{rf}\boldsymbol{\sigma}_\mathbf{z} \tag{9}$$

We note that in this frame the rotation axis of the drive field precesses at a speed determined by the detuning $\mathit{\Delta}_\mathrm{rf,dd}$. Assuming sharp control pulses, we approximate the expression for the drive field using only the starting time $\mathrm{t_i}$ of each sharp pulse

$$\hat{\mathit{\Omega}}_\mathrm{dd} \approx \mathit{\Omega}_\mathrm{dd}\cos\left(\phi_\mathrm{dd} + \mathit{\Delta}_\mathrm{rf,dd}t_i\right)\boldsymbol{\sigma}_\mathbf{x} + \mathit{\Omega}_\mathrm{dd}\sin\left(\phi_\mathrm{rf} + \mathit{\Delta}_\mathrm{rf,dd}t_i\right)\boldsymbol{\sigma}_\mathbf{y} + \mathit{\Delta}_\mathrm{rf}\boldsymbol{\sigma}_\mathbf{z} \tag{10}$$

In this way, we gain two unified Rabi frequencies $\hat{\mathit{\Omega}}_\mathrm{rf}, \hat{\mathit{\Omega}}_\mathrm{dd}$ which we apply for the respective pulses.

$$\hat{\mathit{\Omega}}_\mathrm{u} = \begin{cases} \hat{\mathit{\Omega}}_\mathrm{rf} & \text{for signal pulses} \\ \hat{\mathit{\Omega}}_\mathrm{dd} & \text{for drive pulses} \end{cases}$$

The total time evolution of the spin with initial state $|0\rangle$ under a sequence of pulses $\{U_0(t_0), \dots, U_n(t_n)\},\ t_n > t_0$ is then given by

$$|\Psi_\mathrm{f}\rangle = \prod_{i=n}^{0}\hat{U}_i\,|0\rangle$$

Supplementary Note 4: Robust Pulse Sequences

As shown in the main text, long sequences are favorable for best sensitivity. Since T_2^* sets an upper limit to the pulse spacing τ, this requires application of as many π pulses in the strong dressing sequence as possible. Owing to their capability to correct for pulse errors and environmental fluctuations, dynamical decoupling (DD) protocols are an established technique for implementing such long control pulse trains.

In principle, all DD protocols that contain π pulses with a carrier phase orthogonal to the phase of the signal are compatible with our RF sensing scheme (see discussion of the sensitivity function in Supplementary Note 2). However, if phase control of the signal field is not assumed, an additional constraint on the periodicity of the control pulses arises: Since phase switching is effectively obtained by a constant detuning Δ, such orthogonal pulses (e.g. π_{X} for a signal phase Y) must be equally spaced in the control sequence. Consequently, XY8 cannot be applied under this assumption.

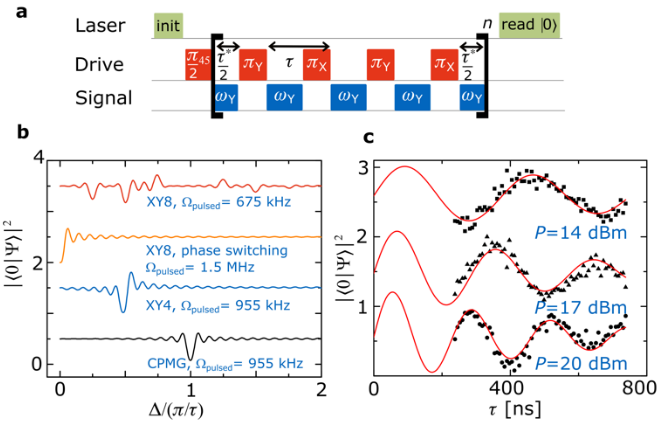

Supplementary Figure 3 – Influence of dynamical decoupling dressing sequence. (a) XY4 sequence employed to record the data shown in (c) $\pi_{45}/2$ denotes a carrier phase of $\phi = \pi/4$ (b) Simulation of the spectral response to sensing sequences with different decoupling protocols (reproduced from Fig. 3e *with added data). All simulations use 24 π pulses with a fixed pulse spacing τ=43.6*ns. *The signal Rabi frequency was set to $\Omega_{rf} = 1.5 * 10^6\ Hz$, yielding effective Rabi frequencies $\Omega_{XY8,ps} = \Omega_{rf}$, $\Omega_{XY8} = \frac{\sqrt{2}}{\pi}\Omega_{rf} \approx 0.45\Omega_{rf}$, $\Omega_{XY4} = \Omega_{CPMG} = \frac{2}{\pi}\Omega_{rf} \approx 0.64\Omega_{rf}$. (c) Rabi oscillations measured for distinct signal powers P and using 18 π pulses along the hyperbola defined by $\Delta = \frac{\pi}{2\tau}$. Data traces in (b+c) have been shifted vertically by 1.0 for better comparison.*

As shown in Supplementary Figure 3b and Fig. 3e of the main text, a different spectral response arises depending on the distinct DD protocol used: XY8 without phase switching of the weak signal (red curve) shows a complex sideband structure with multiple strongly damped Mollow resonances, reflecting the different periodicities in the sequence. On the most pronounced resonance at $\Delta = \pi/2\tau$, a reduced effective Rabi frequency of $\Omega_{\mathrm{XY8}} = \frac{\sqrt{2}}{\pi}\Omega_{\mathrm{rf}}$ is observable. However, by adding a suitable phase switching (orange curve, see Fig. 4a for the pulse protocol), we can make the central Mollow peak sensitive to the weak field, i.e. drive weak Rabi oscillations with frequency Ω_{rf} at $\Delta = 0$.

In the case of a CPMG dressing sequence (protocol shown in Fig. 3b), π pulses with a phase orthogonal to the signal field are equidistantly spaced. Consequently, weak Rabi oscillations with effective frequency $\Omega_{\mathrm{CPMG}} = \frac{2}{\pi}\Omega_{\mathrm{rf}}$ can be driven in the Mollow resonance at detuning $\Delta = \pi/\tau$ (black curve in Supplementary Figure 3b).

The effect of the spacing between orthogonal pulses becomes clear when we compare this to the response to a XY4 protocol as shown in Supplementary Figure 3a. Here, only the phase of every

second π pulse is orthogonal to the signal phase. As a result, we observe (blue curve in Supplementary Figure 3b) that the Mollow resonance is shifted to $\Delta = \pi/2\tau$, that is half of the detuning at which the CPMG resonance appears. By contrast, the effective Rabi frequency $\Omega_{\mathrm{XY4}} = \frac{2}{\pi}\Omega_{\mathrm{rf}}$ is identical.

To confirm the behavior observed in our simulations, we also obtained experimental data for XY4, where we controlled the detuning such that Rabi oscillations along the hyperbola $\Delta = \pi/2\tau$ could be measured (Supplementary Figure 3c). The data shows excellent agreement with the predicted evolution, confirming the applicability of our model.

Supplementary Note 5: Continuous signals

We have verified that high-frequency sensing remains sensitive to continuous signals covering the π-pulses of the strong drive. These experiments have been performed analogous to the experiments described in Fig 4, with results presented in Supplementary Figure 4. In agreement with theory, continuous signals can drive Rabi oscillations on a pulsed Mollow resonance. While this general statement applies to any dynamical decoupling sequence with a suitable resonance, we have observed that the contrast of the resulting oscillations is higher for sequences with a high degree of robustness against experimental fluctuations, such as XY4.

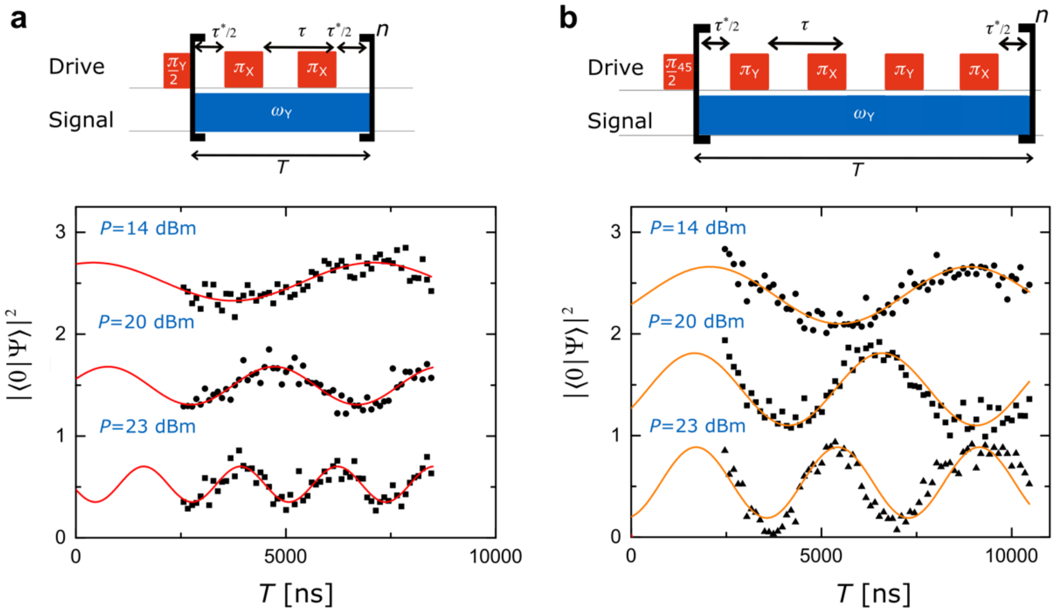

Supplementary Figure 4 - Sensing of continuous signals. (a) Sensing by a CPMG sequence. (b) Sensing by a XY4 sequence. Both experiments reveal Rabi oscillations much slower than $T_2^* \approx 1\mu s$. t_{RF} denotes the full duration of the signal. Signal contrast is higher for XY4 (b), since this sequence is more robust against fluctuations. Measurements at different powers have been shifted against each other by an offset of 1.0. $\pi_{45}/2$ denotes a carrier phase of $\phi = \pi/4$
